# Aroma characterization and consumer acceptance of four cookie products enriched with insect (*Ruspolia differens*) meal

**DOI:** 10.1038/s41598-023-38166-x

**Published:** 2023-07-10

**Authors:** Brian O. Ochieng, Joseph O. Anyango, John M. Nduko, Cynthia M. Mudalungu, Xavier Cheseto, Chrysantus M. Tanga

**Affiliations:** 1 International Centre of Insect Physiology and Ecology (Icipe), P.O. BOX 30772, Nairobi, 00100 Kenya; 2 Department of Dairy and Food Science and Technology, Egerton University, P.O. Box 536, Njoro, 20115 Kenya

**Keywords:** Entomology, Chemical ecology

## Abstract

This research aims to advance knowledge on the impact of four processing methods on volatile compounds from insect-based baked products (cookies) to provide insights on consumer acceptance. Samples were exposed to double step enzyme digestive test, volatiles characterized through headspace analysis, while semi-trained panelists were recruited for the sensory test. Blanched and boiled samples of *R. differens* had considerably higher digestibility (83.42% and 81.61%, respectively) (*p* < 0.05) than toasted and deep-fried samples. Insect-based cookie products integrated with blanched and boiled *R. differens* meal expressed higher digestibility (80.41% and 78.73%, respectively) that was comparable to that of commercial cookie products (control cookies-CTRC with 88.22%). Key volatile compounds common between the various cookie products included, nonanal, octanal, methyl-pyrazine, hexanal, tetradecane, 2-pentylfuran, 2-heptanone, 2*E*-octenal, 2*E*-heptenal and dodecane. Among the volatile compounds, pleasant aromas observed were 2*E*,4*E*-dodecadienal, pentanal, octanal, methyl pyrazine, furfurals, benzaldehyde, and 2-pentyl furan, which were more pronounced in cookies fortified with boiled, toasted and deep-fried *R. differens* meal. There was a greater resemblance of sensory characteristics between control cookies and those fortified with deep-fried *R. differens*. These findings underscore the significant influence of aroma compounds on consumer acceptability and preference for insect-based baked food products, which allows for future process-modification of innate aromas of insect-based meals to produce high-valued pleasant consumer driven market products.

Edible insects portray enormous nutritional potential accentuating excellent sources of proteins, lipids, certain vitamins, and minerals, such as calcium, iron, or zinc^1^ and healthy unsaturated fatty acids^2^. This rich nutritional profile has attracted the attention of researchers and the food industry for their possible application in the development of foods with improved nutritional qualities to promote human nutrition. This initiative has however, experienced challenges with regards to consumer acceptance of insect-based products^3^, characterized by food neophobia and disgust^4^. Moreover, even if the insect-based products are accepted, their nutritional copiousness does not necessarily signify their high digestibility^5^. It is therefore paramount to understand the bio-accessibility of the proteins, especially after processing to guarantee nutritional benefits to the consumers.

Digestibility determines whether physiologically active and nutritionally valuable molecules are released from the food matrix into the gastrointestinal tract, making them available for intestinal absorption^5^. Despite edible insects’ high digestibility of 76–98%^6^, factors such as species differences, chitin levels and processing techniques significantly influence it^7^. From literature, toasted and dried *R. differens* demonstrated higher digestibility as opposed to termites^8^, boiled and oven cooked mealworms displayed higher digestibility^9^ and toasting of mopane worms remarkably declined their digestibility^10^.

Food neophobia is a personality trait characterized by the avoidance of novel or unfamiliar meals, whereas disgust is associated with implicit attitudes that are impacted by people's implicit associations with a food's disgust-inducing characteristics^4^. Of great influence to acceptability of edible insects is disgust, as it has been cited the major deterrent factor to entomophagy adoption^11–13^. Disgust is provoked by sensory characteristics such as aroma, texture and general appearance^3^. Development of insect-based products with modified sensory properties aligned to the gastronomic customs of consumers has enhanced consumer preference and familiarity to these products to some extent^4^. However, reduced ranking of sensory attributes has been witnessed when the insect-based products are compared against their insect-free counterparts^14–16^.

Distaste of insect-based products, galvanized by aroma and flavours, is the key reason behind consumer prejudice of these products marked by reluctant consideration into diets^17,18^. Flavours in edible insects are derived from the pheromone on their surfaces, the environment where they grow or are bred, the feeds they feed/fed on and fermentation products^19–21^. Therefore, research directed towards the modification of these sensory properties of edible insects into consumer-pleasant properties in a bid to comply with the FAO advocacy for insects is pertinent.

A distinctive study by Ssepuuya et al.^22^ expressed boiling *R. differens* to enhance the levels of aroma compounds hexanal and 2-pentylfuran and further toasting to markedly elevate the levels of heptanal, octanal, nonanal, 2-heptanone, 2-nonanone, 2-decanone and limonene. Cheseto et al.^23^ equally reported a number of ketones and aldehydes in cookies baked with different insect oils including *R. differens* oil. Considerable concentrations of these chemical compounds were associated with increased consumer acceptability.

This study purposely sought to integrate *R. differens* flours processed through blanching, boiling, toasting and deep-frying into cookies to assess how they impact protein digestibility, aroma compounds and sensory characteristics. Cookies are cereal-based bakery products that are quite famous and well-liked all around the world, and their formulations with low concentrations of insect flours have proven to be better acceptable and familiarized by consumers^24^.

## Results

### In vitro protein digestibility of processed *R. differens* and the respective cookies

**Figure 1:**
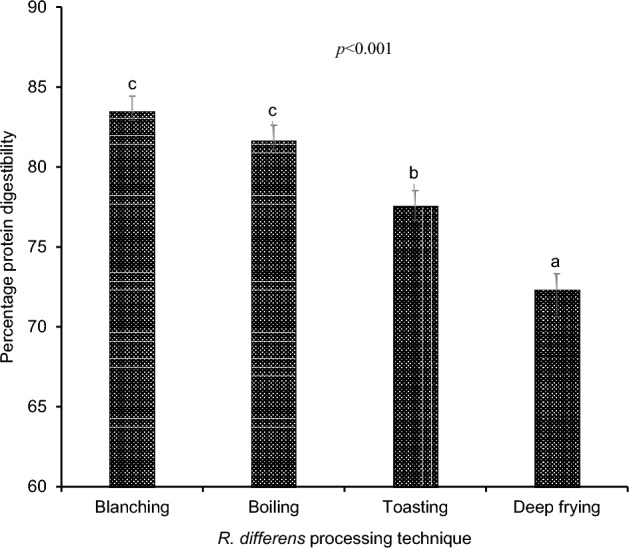
Bar chart showing the variation of in vitro protein digestibility (%) of differently processed *R. differens*. Error bars indicate standard deviation of the mean. Bars with same small letter are not significantly different.

**Figure 2:**
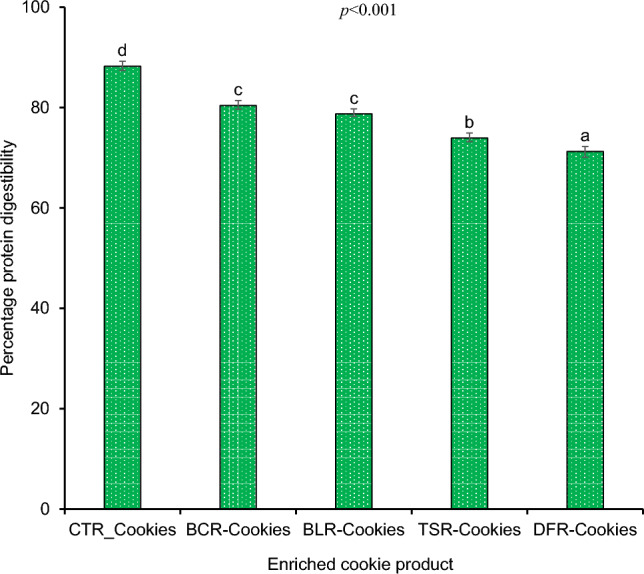
In vitro protein digestibility (%) of the processed R. differens based cookies. Bars with same small letters following each other are not significantly different (*p* < 0.05). *CTRC* Control cookies with eggs; *BCRC* Blanched *R. differens*-based cookies; *BLRC* Boiled *R. differens*-based cookies; *TSRC* Toasted *R. differens*-based cookies; *DFRC* Deep fried *R. differens*-based cookies.

The percentage in vitro protein digestibility of *R. differens* after exposure to different processing methods are illustrated in Fig. 1. Both blanched and boiled *R. differens* had considerably higher protein digestibility (*p* < 0.05) than toasted and deep-fried samples. For the cookies, higher digestibility (*p* < 0.05) was depicted in the CTRC with DFRC recording the lowest (Fig. 2). However, there was no discernible difference in digestibility between BCRC and BLRC.

### Sensory evaluation of the cookies

**Figure 3:**
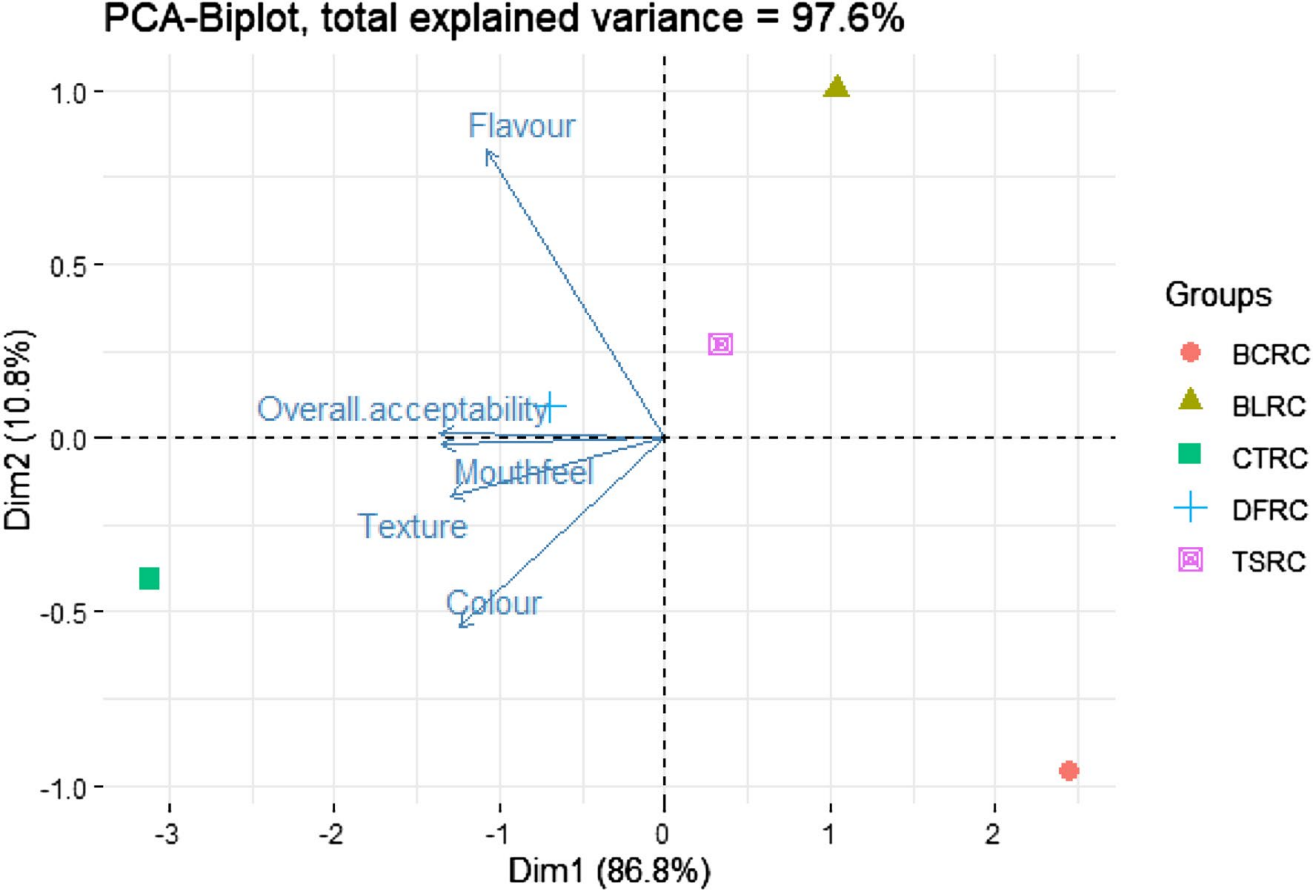
Principal component analysis (PCA) biplot showing the variation of sensory attribute scores among the cookies enriched with differentially processed R. differens. CTRC = Control cookies with eggs; BCRC = Blanched R. differens-based cookies; BLRC = Boiled R. differens-based cookies; TSRC = Toasted R. differens-based cookies; DFRC = Deep fried R. differens-based cookies.

The sensory attributes; colour (F_(5,852)_ = 11.8, *p* < 0.001), flavour (F_(5,852)_ = 14.3, *p* < 0.001), mouthfeel (F_(5,852)_ = 6.2, *p* < 0.001), texture (F_(5,852)_ = 7.8, *p* < 0.001) and overall acceptability (F_(5,852)_ = 14.7, *p* < 0.001) varied significantly across the five cookie types (Table 1**)**. The texture ratings of CTRC samples were significantly (*p* < 0.05) higher than the other cookie types with the colour and overall acceptability ratings being significantly (*p* < 0.05) higher than BCRC, BLRC and TSRC only. The consumers rated the colour, texture, flavour, and overall acceptability of both CTRC and DFRC equally. In addition, when compared to other cookies, BCRC recorded significantly (*p* < 0.05) lower preference of flavour attributes. A two-dimensional Principal Component Analysis (PCA) explaining 97.6% of the variation differentiated the cookies based on their sensory scores and portrayed the correlation between the sensory characteristics of the cookies (Fig. 3). The PCA revealed strong positive correlation between mouthfeel and overall acceptability.

**Table 1:** Mean sensory scores of the cookies enriched with differently processed *R. differens* flours.

Cookies	Colour	Flavour	Mouthfeel	Texture	Overall acceptability
CTRC	4.47 ± 0.92^c^	3.95 ± 1.04^b^	4.22 ± 0.98^c^	4.07 ± 0.94^c^	4.24 ± 0.97^d^
BCRC	3.83 ± 1.03^ab^	3.17 ± 1.29^a^	3.68 ± 1.12^a^	3.55 ± 1.07^ab^	3.58 ± 0.95^ab^
BLRC	3.68 ± 1.11^a^	3.83 ± 1.02^b^	3.80 ± 1.02^ab^	3.64 ± 0.91^ab^	3.75 ± 0.95^bc^
TSRC	3.92 ± 0.86^ab^	3.72 ± 1.02^b^	3.91 ± 1.07^abc^	3.66 ± 1.01^ab^	3.85 ± 0.94^bc^
DFRC	4.15 ± 0.89^bc^	3.81 ± 0.93^b^	4.04 ± 0.94^bc^	3.69 ± 1.05^b^	3.97 ± 0.86^ cd^

Values are presented as means ± SD of triplicate determinations. Means in the same raw followed by same small superscript letters are not significantly different at *p* < 0.05.

*CTRC* Control cookies with eggs; *BCRC* Blanched *R. differens*-based cookies; *BLRC* Boiled *R. differens*-based cookies; *TSRC* Toasted *R. differens*-based cookies; *DFRC* Deep fried *R. differens*-based cookies.

### Volatile organic compounds of the cookies

**Figure 4:**
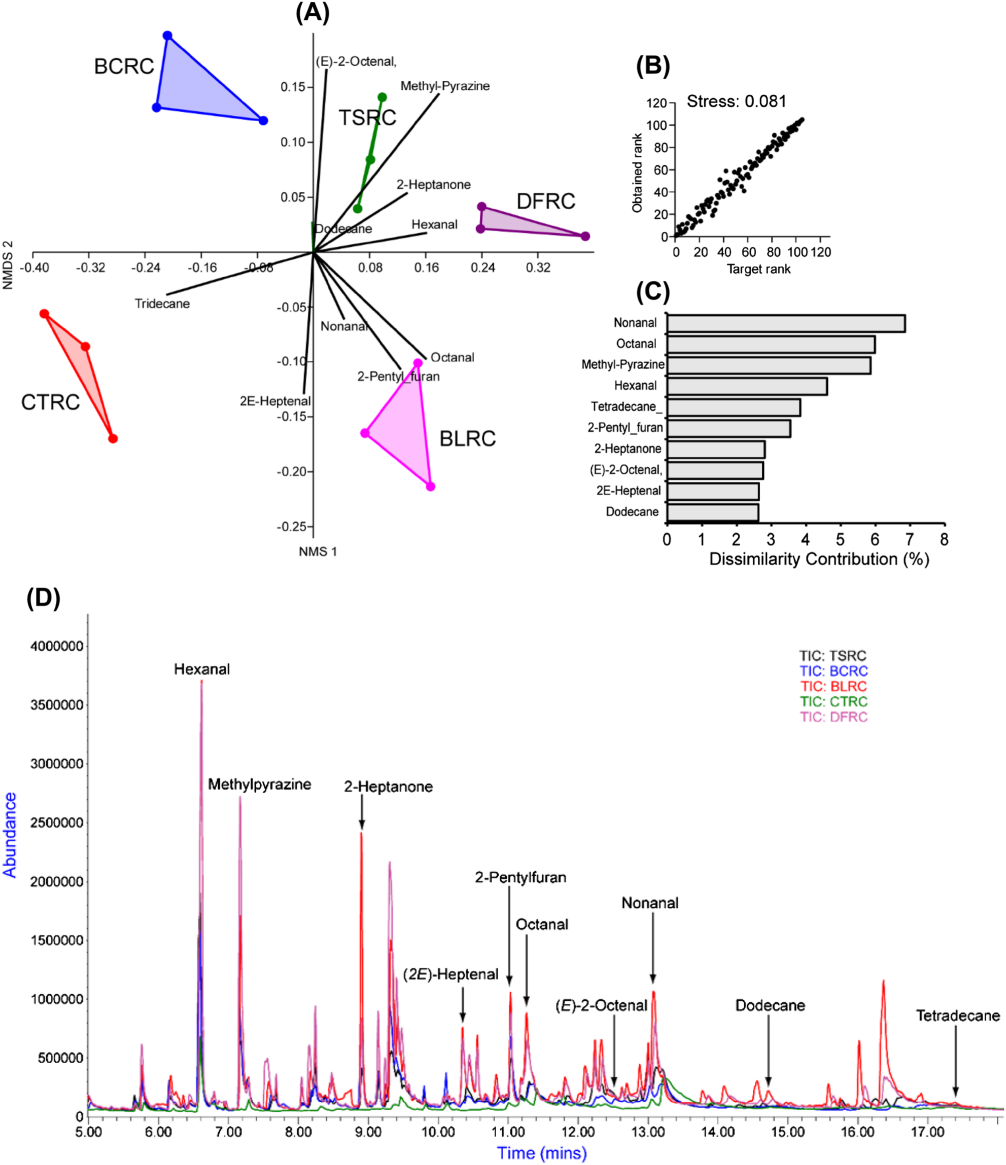
(**A**) Non-metric multidimensional scaling plot (NMDS) clustering the different *R. differens*-based cookies based on the type of volatile they emit, analysis of similarities (ANOSIM). (**B**) Shepard plot showing the great ordination of the NMDS analysis (stress value < 0.1). (**C**) Histogram displaying the contribution of the 10 most important volatiles to the differentiation of all the different enriched cookies. CTRC = Control cookies with eggs; BCRC = Blanched *R. differens*-based cookies; BLRC = Boiled *R. differens*-based cookies; TSRC = Toasted *R. differens*-based cookies; DFRC = Deep fried *R. differens*-based cookies. (**D**) Overlayed Total ion Chromatogram (TIC) indicating some of the identified violates in C. Each cookie chromatogram is represented by a different colour.

Chemical characterization of headspace components of the cookies revealed 79 major distinct volatile organic compounds (VOCs) presented in supplementary Table 1 (Table S1). The VOCs dominantly comprised of hydrocarbons, aldehydes, monoterpenes, and ketones. The stress value of < 0.1 (0.081) from the Non-Metric Multidimensional Scaling (NMDS) ordination (Fig. 4A) indicated a good representation of the dissimilarities and correct grouping of the VOCs from the cookies enriched with differently processed *R. differens* (Fig. 4B). The major compounds that led to the differentiation of the cookies enriched with differently processed *R. differens* are nonanal, octanal, methyl-pyrazine, hexanal, tridecane, 2-pentylfuran, 2-heptanone, (*E*)-2-octenal, (*E*)-2-heptenal, and dodecane (Fig. 4A and C) with their respective total ion chromatogram presented in Fig. 4D and peak areas presented in Table 2.

**Table 2:** Key volatile compounds that led to the differentiation of insect-fortified cookies as identified by non-metric multidimensional scaling plot.

^a^tR (min)	^b^RI	Compound	^c^M + ion	^d^Fragment ions	Compound class	^e^Cookie types					Descriptors
						CTRC	BCRC	BLRC	TSRC	DFRC	
6.59	793	Hexanal	110.1	44.1, 56.1, 72.1, 82.1	Aldehyde	2.6 ± 0.4	3.8 ± 1.1	5.6 ± 3.5	4.9 ± 0.8	5.9 ± 3.4	Green apple, grassy
7.18	815	Methylpyrazine	94.1	67.1	Pyrazine	0.2 ± 0.1	3.4 ± 1.0	1.8 ± 2.7	2.6 ± 0.2	5.9 ± 1.4	Nutty, cocoa, roasted meat
8.90	882	2-Heptanone	114.1	43.1, 58.1, 71.1	Ketone	1.7 ± 0.8	1.7 ± 0.8	2.2 ± 2.6	1.3 ± 0.3	1.7 ± 0.5	Sour, brown, fruity, sweet
10.35	944	2*E*-Heptenal	112.1	40.0, 55.1, 70.1, 83.1, 95.0	Aldehyde	0.5 ± 0.2	1.3 ± 0.1	2.7 ± 0.7	1.4 ± 0.4	2.3 ± 0.2	Almond-like, fatty
11.03	974	2-Pentylfuran	138.1	53.1, 81.1, 95.1	Furan	1.3 ± 0.2	2.0 ± 1.0	4.6 ± 4.6	1.9 ± 0.5	2.9 ± 0.3	Sweet, woody, almond-like, baked bread
11.32	988	Octanal	128.1	43.1, 56.1, 69.1, 84.0	Aldehyde	2.1 ± 1.5	0.6 ± 0.1	5.3 ± 4.4	3.6 ± 0.1	4.9 ± 0.2	Fruity, nutty
12.55	1059	(*E*)-2-Octenal	126.1	41.1, 55.1, 70.1, 84.0, 97.1	Aldehyde	–	0.9 ± 0.1	–	2.9 ± 0.2	–	Fatty, grass, soap, beany, soy, nutty
13.11	1092	Nonanal	142.2	43.1, 57.1, 70.1, 82.1, 98.1, 114.1	Aldehyde	7.3 ± 0.6	2.1 ± 0.1	3.6 ± 1.8	4.8 ± 1.7	7.5 ± 0.7	Dusty, nutty, cleaner
14.77	1190	Dodecane	170.2	43.1, 57.1, 71.1, 84.0	Hydrocarbon	1.8 ± 0.2	0.3 ± 0.1	0.7 ± 0.2	2.6 ± 3.3	1.0 ± 0.1	–
17.50	1377	Tetradecane	198.2	43.1, 57.1, 71.1, 84.0, 99.1, 127.1, 155.1	Hydrocarbon	3.0 ± 0.4	1.6 ± 0.2	1.7 ± 0.4	0.2 ± 0.0	1.4 ± 1.0	–

^e^values (Mean ± standard deviation) acquired from GC–MS peak responses of cookies using three replicates and expressed as peak area/10^7^.

^a^Retention time in minutes.

^b^Retention Index.

^c^Molecular ions mass.

^d^Fragment ions mass.

*CTRC* Control cookies with eggs; *BCRC* Blanched *R. differens*-based cookies; *BLRC* Boiled *R. differens*-based cookies; *TSRC* Toasted *R. differens*-based cookies; *DFRC* Deep fried *R. differens*-based cookies. The odour descriptions are retrieved from literature^25–27^.

## Discussion

Protein digestibility is a measure of a protein's or its structural subunits' (peptides and amino acids) bio-accessibility in the gut relative to what is consumed. The processed insects' in vitro protein digestibility varied from 72.32 to 83.42%, which corroborates prior reports indicating 76–96% in edible insects^28^. Edible insect proteins are slightly less digestible than eggs (95%) and beef (98%) but are more digestible than plant proteins^20^. In the present study, the blanched *R. differens* expressed highest digestibility followed by boiled, toasted and then deep-fried *R. differens* thereby exposing the impactful consequences of their respective processes. Thermal processing has been reported to either increase or decrease protein digestibility depending on processing conditions and circumstances. Kinyuru et al.^8^ found that toasting and drying reduced protein digestibility of *R. differens* while unaltering that of edible winged termites (*Macrotermes subhylanus*).

Denaturation temperatures improve native protein digestibility by unfolding the polypeptide chain and making the protein more accessible to digestive enzymes^8^. The decreased protein digestibility exhibited in toasted and deep-fried samples may be due to exposure of proteins to dry heat, which promotes the formation of disulphide linkages, hampering digestive enzymes accessibility. Furthermore, the low moisture and high temperature conditions characterizing toasting and deep frying processes, intensified Maillard reactions which may have utilized available proteins and amino acids, thereby reducing the amount of digestible proteins^29^. Also, the greater fat content of deep-fried and toasted *R. differens* compared to blanched and boiling samples^30^ may have promoted the formation of protein-lipid oxidation products complexes posing a hindrance to enzyme-protein accessibility^5^. Antinutrients (tannins and phytates), chitin, and the experimental approach used in terms of enzymes applied could all play a role in explaining the differences in digestibility identified in edible insect proteins^7^. Although there is still inadequate information on the anti-nutrient content of *R. differens*, analysis of its closely related species *S. prasiniferum* and *C. trachypterus* revealed that their levels were within acceptable ranges^31^ hence, may have not significantly influenced the digestibility. In this respect, due to its soft body nature, *R. differens* has a low chitinous content, which could have had little impact on digestion^5^.

The protein digestibility of the enriched cookies paralleled the pattern observed in their processed *R. differens* counterparts. As a result, it is possible that the elements that limit or enhance digestibility in processed *R. differens* also played a role in the protein digestibility of the cookies. This is consistent with findings made by Akullo et al.^29^ which ascribed differences in protein digestibility of crackers to the influence of termite processing conditions. In other studies, increasing the quantity of Bambara ground nut flours with high protein digestibility boosted the protein digestibility of non-wheat cookies^32^. Contrastingly, the enhanced cookies' protein digestibility was marginally lower than that of their processed *R. differens* counterparts. This could possibly be attributed to the processed *R. differens* having a higher digestible protein content (7.8–44.7%)^30^ compared to the cookies (nutrients dilution effect). Other factors such as such as physical parameters and enzyme(s) have been shown to affect digestibility. For instance, Abdel-Aal^33^ found that biscuits had improved digestibility in a two-step enzyme metabolism but decreased digestibility in a one-step enzyme metabolism. The former was associated to increased protein accessibility by enzymes during baking, while the latter was linked to a pH change caused by the biscuit mix's buffering capacity. The inclusion of eggs in the formulation with the omission of the insect flours resulted in a higher digestibility of control cookies (CTRC). Eggs are known to have a high protein digestibility (95%)^20^. This is explained by their lack of chitin, which correlates adversely with protein digestibility^7^ as well as the presence of highly soluble proteins.

Despite the fact that a significant level of insect familiarity has been gained as a result of increased awareness and incorporation into modern food products, a significant number of consumers remain opposed to the idea. Authors have established that there is a general trend of a negative association between increasing levels of insect incorporation into products and their acceptability^15,34–38^. Contrary to Bawa et al.^39^, Ojinnaka et al.^37^ and Adeboye et al.^34^ reporting a significant difference in flavour perception of 10% insect enriched cookies compared to the control, the current study found no significant differences in BLRC, TSRC, and DFRC, but not BCRC (Table 1). This may be attributed to the raw insects' original terrible flavours being transformed into new, more attractive flavours as a result of the processing. BCRCs were developed using blanched *R. differens*, a process characterized by short-lived hot water treatment, which may have been inadequate for total transformation of the flavour compounds. Consumers' aversion to edible insects has been highlighted as the most common barrier to their acceptance. The primary elicitors have been identified as sensory qualities of insects such as flavour, appearance, and texture^40,41^ and determines whether an insect-based product is acceptable or not^37^. However, flavours were very weakly correlated to overall acceptability in this study (Fig. 3B), most likely due to the unpleasant flavours, which were not considered to gauge the acceptability. CTRC's colour rankings differed greatly from BCRC, BLRC, and TSRC. This could be due to the demonstrated higher levels of accessible proteins, peptides, and amino acids in blanched, boiled, and toasted *R. differens*^30^ catalyzing Maillard reactions^37,42^, responsible for darker colours in BCRC, BLRC and TSRC (Fig. 1). Notably, there was no statistically significant difference in colour scores between CTRC and DFRC, which may be due to the deep-frying temperatures degradation of proteins and amino acids in the *R. differens*^30^ employed in the DFRC formulation. The exoskeleton of the ground *R. differens* integrated in the insect-based cookies explain the significant variations in texture scores between the CTRC and the insect-based cookies^37^. The mouthfeel of the cookies substantially correlated positively with overall acceptance (R = 0.99), indicating that the majority of the panellists relied on physical sensation of the cookies while in the mouth to judge their general acceptability. CTRC was the most popular choice in general hence consistent with findings from other researchers^15,29,37^. The use of a 5-point hedonic scale was adopted due to its less complexity and suitability for naïve assessors. However, future studies should employ 9-point hedonic scales for comprehensive product sensory evaluations and deduction of consumer trends.

Volatile organic compounds (VOCs), which contribute to aroma, flavour, and taste, are one of the features that influence the perception and acceptance of foods, including edible insects. Acids, alcohols, aldehydes, alkenes, amines, terpenes, ketones, and esters are among the aroma chemicals previously discovered in insects^19,22,23^ which translate to savory, umami, buttery, meaty, bacony, sweet, herbal, or fruity flavours^43^. The main VOCs that contributed significantly to cookie difference are consistent with the profiles found in boiled and roasted *R. differens*^22^ and *R. differens* oil^23^. The higher concentrations of hexanal and 2-pentylfuran in BLRC cookies prepared with boiled *R. differens* underpins the findings by Ssepuuya et al.^22^ revealing that boiling *R. differens* enhances the two compounds. The detection of limonene in all the cookies and 1-heptanol in the BLRC is in agreement with a previous study which found limonene and heptanol as the predominant volatiles in raw *R. differens*^22^. Similarly, the pronounced levels of hexanal and 2-pentylfuran in BLRC are consistent with previous research that found the two aroma compounds to be the most prevalent VOCs in boiled *R. differens*^22^. High methyl pyrazine concentrations associated with TSRC and DFRC (formulated with toasted and deep-fried *R. differens*, respectively) maybe hypothesized to emanate from oxidation decarboxylation of reducing sugars and amino acids during frying process to generate Strecker aldehydes and α-amrinones. The resultant compounds subsequently condense to form alkane pyrazines, hence serving as dominant aroma compounds in toasted and deep-fried *R. differens* and in their respective cookie products^44^.

Unsaturated fatty acid breakdown has been reported to produce 2*E*,4*E*-decadienal, and 2*E*-heptenal^45^. In this study, the higher 2*E*,4*E*-decadienal found in TSRC and DFRC could be attributed to their emergence during high-temperature toasting and deep-frying of *R. differens*. BCRC were characterized with low concentrations of nonanal, hexanal, 2*E*,4*E*-dodecadienal, pentanal, and octanal are aldehydes which are associated with fat, meaty flavour, nutty, sweet, and almond-like aroma^26,46^ and methyl pyrazine, furfurals, benzaldehyde and 2-pentyl furan which are associated with desirable flavours; nutty, cocoa, roasted meat, almond-like and sweet. This may have contributed to the low sensory scores regarding flavour of the BCRC compared with the other cookie types. BCRC were formulated with *R. differens* processed by blanching, an ephemeral processing technique, which may have resulted in insufficient chemical interactions to produce adequate flavour profiles. This is apparent from Fig. 4A displaying no VOC associated with BCRC from the identified influential profiles in Fig. 4C. In another study, fermentation of *Allomyrina dichotoma* larvae with *Saccharomyces cerevisiae* significantly reduced indole, a faecal odour compound, while simultaneously introducing new aroma compounds such as 2-undecanone, 2-methyl-1-butanol, 2-nonanone, 3-methyl-1-butanol, isopentyl acetate, and ethyl acetate and enhancing others^47^. Therefore, edible insects processing can be a prospective strategy adoptable to manipulate insect aroma from native and undesirable profiles to new pleasant profiles in order to advance entomophagy. The current study is by no means exhaustive. Therefore, future use of other techniques such as Gas chromatography–olfactometry-mass spectrometry for identification of aroma active compounds would be crucial.

## Conclusion

The high levels of protein digestibility observed in *R. differens* meals was clearly mirrored in the value-added cookie products. Most of the volatile compounds produced by the baked cookie products fortified with *R. differens* meal were associated with attractive aroma. These findings suggest that different processing conditions could be used to obtain diversified insect-based food products contributing to increased consumer satisfaction. This groups of volatile compounds; acetoin, pyrazine, (*E*)-3-penten-2-one, 2,3-butanediol, 2,3,5-trimethyl hexane, methylpyrazine, furfural, 2,4-dimethyl heptanone, 2-heptanone, benzaldehyde, eicosane and (2*E*,4*Z*)-decadienal in *R. differens* fortified cookies highlights the unique specificity of these products from conventional cookie bakery product. Given that aroma and flavour of novel food products could be a deterrent factor to consumers, further investigations are needed for quality control.

## Materials and methods

### Acquisition and processing of *R. differens*

Fresh and sorted *R. differens* of 20 kg, with the ovipositors, appendages and wings removed, were purchased from Masaka (0°20′ 28.0′′ S 31° 44′10.0′′ E) and Kampala (0.3476° N, 32.5825° E), Uganda in 2019. The samples were packed into sterile sample collection plastic containers, placed in cool boxes, covered with flaked ice (4–7 °C) and transported to International Centre of Insect Physiology and Ecology (*icipe*) laboratory. Blanching, boiling, toasting and deep-frying were adopted for processing 700 g each of *R. differens* and oven-dried (SDO-225, Wagtech International, Thatcham, UK) at 60 ℃ for 24 h to a moisture content of < 15% according to procedures delineated by Ochieng et al.^30^. The samples were milled using a three-speed Waring laboratory blender, (Camlab, Over, UK) and screened through a 0.1 mm stainless steel laboratory sieve. They were then subsequently vacuum packed in sterile zip loc bags, labelled accordingly and temporarily stored at −4 ℃ awaiting formulation.

### Formulation and baking of cookies

**Figure 5:**
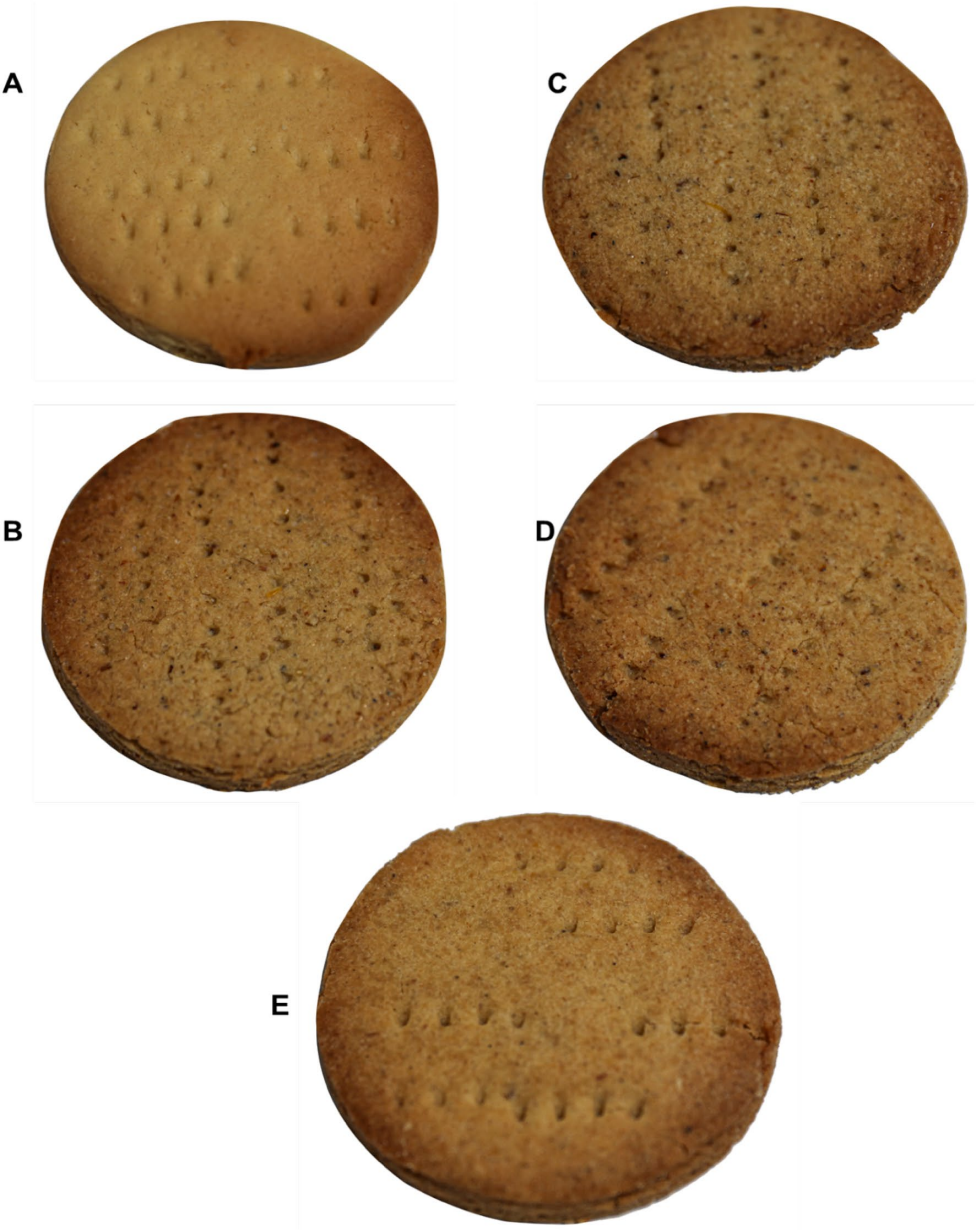
Cookies made from differently processes *R. differens*. (**A**): CTRC-Control (i.e., without *R. differens* meal) cookie; (**B**): BCRC-Blanched *R. differens*-based cookie; (**C**): BLRC-Boiled *R. differens*-based cookie; (**D**): TSRC-Toasted *R. differens*-based Cookie and (**E**): DFRC-Deep-fried *R. differens*-based cookie.

Cookies enriched with the processed *R. differens* as well as the control were formulated according to a method described by Aziah et al.^48^, with a few modifications. Wheat was substituted with the processed *R. differens* at 10% (w/w) based on a consistently demonstrated marginal acceptability of sensory characteristics of bakery products previously formulated with insects flours at 10% inclusion level^15,34–38^. Cookies with no insects added, contained eggs, serving as the control. About 172.2 g of sugar and 3.4 g of salt were sieved and mixed with 408.2 g of wheat flour, already premixed with food grade improvers, for 5 min. Approximately 172.2 g of shortening was added and mixed in a bakery mixer (BJY-BM10, Berjaya, Malaysia) for 15 min to produce a creamy mixture. About 84 g of whisked eggs (for control) or processed *R. differens* flours was added and mixed for another 10 min. The mixture was then hand-kneaded for 5 min to obtain a firm consistent dough of approximately 180 g each. The dough was rolled out on a wooden board using a rolling pin to a thickness of 5 mm and cut into 5 cm diameter circles. The cut-out cookie doughs were arranged on greased baking trays at 50 mm apart and baked for 15 min at 180 °C, 30% dryness in a preheated oven (BISTROT 665; BestFor®, Ferrara, Italy). Approximately 180 cookies were prepared from each formulated dough with each cookie type represented in Fig. 5. Three cookies, from each treatment were randomly selected and immediately taken for headspace volatiles trapping. Cookies (143) from each dough, intended for sensory study, was packed in 2-inch mini plastic zip loc bags and coded accordingly. The remaining cookies were kept in a cold room at −10 ℃ for a successive digestibility test.

### In vitro protein digestibility of the processed *R. differens* flours and their respective cookies

Determination of in vitro protein digestibility of the processed *R. differens* and their respective cookies were conducted according to Chavan et al.^49^ and modified by Wang et al.^50^. Samples of 1 g each were weighed into 50 mL centrifuge tubes, which were then filled with 20 mL of 0.10 M HCl. Likewise, 50 mg of pepsin (Porcine gastric mucosa, ≥ 250 units/mg solid, Sigma-Aldrich) suspended in 1 mL of 0.01 M HCl were added and mixed. The mixture was then gently shaken for 3 h in a water bath shaker (GYROMAX, Amerex Instruments, Inc, CA, USA) at 37 °C. Subsequently, a mix of 10 mL distilled water and 10 mL of 0.1 M phosphate buffer (pH 8.0) containing 5 mg of trypsin (porcine pancreas, lyophilized powder, BioReagent, 1000–2000 BAEE units/mg solid, Sigma-Aldrich) was introduced, and the mixture subjected to a 37 ℃ water bath for 3 h under continuous agitation. Ten millilitres of trichloroacetic acid (TCA) was infused to purposefully terminate the enzymes activity followed by centrifugation (Eppendorf AG, Hamburg, Germany, 2500 g, 20 ℃) at 14 000 rpm for 10 min. The supernatant was discarded, and the residue dried in an oven at 105 ℃ for 3 h. The nitrogen content of dried residue (0.5 g) was measured using the Kjeldahl technique. The difference between the total quantity of protein in the samples and the remaining protein after enzyme digestion was divided by the total protein in the samples to compute the protein digestibility. The protein content of blanched, boiled, toasted and deep-fried *R. differens* (40.1, 43.1, 44.7 and 7.8%, respectively)^30^ and those of cookies; CTRC, BCRC, BLRC, TSRC and DFRC (11.09, 10.90, 10.99 and 6.78%, respectively) were considered as initial protein conents for computation of % digestibility.

### Sensory evaluation of the cookies

The cookies' appearance, taste, mouthfeel, texture, and general acceptability were evaluated for consumer preference. A 5-point hedonic scale was used where; 5 denoted like extremely, 4 denoted like, 3 denoted neither like or dislike, 2 denoted dislike and 1 denoted dislike extremely^51^, the ranking test evaluated differences in intensity of the sensory properties among samples using comparable intervals between the categories. The experiment randomly enrolled a team of 143 semi-trained panellists, comprised of 72 males and 73 females, of age ranging 18–50 years. The panellists were selected based on their experience in food products description and knowledge of cookies. Individual temporary booths made of paperboards for segregation of assessors were equipped with pens and questionnaires for data collection and processing in a sensory laboratory room that almost practically resembled ISO requirements as part of the examinations were set up^52^. The samples (coded as NJM for CTRC, VPK for BCRC, HQT for BLRC, UAL for TSRC and YHP for DFRC) cookie samples were served to the panellists at room temperature. Alongside the samples, the panellists were given a cup of room-temperature clean water for palate cleansing before commencement of the test and between every tasting done. They were instructed to consent to the study, carefully read the instructions and focus on the texture and colour of the cookies first before proceeding to taste. Panellists were required to score the samples against attributes provided in the evaluation forms. The scores were compiled and analyzed.

### Ethical approval

This research was approved by the Institutional Animal Care and Use Committee (IACUC) of Kenya Agricultural and Livestock Research Organization (KALRO)-Veterinary Science Research Institute (VSRI); Muguga North upon compliance with all provisions vetted under and coded: KALRO-VSRI/IACUC028/16032022. This study was reviewed and approved by Egerton University and the National Council for Science Technology and Innovation in Kenya (NACOSTI/P/21/8303). Further, an informed consent was obtained from all the participants and/or their legal guardians via the statement "I am aware that my responses are confidential, and I agree to participate in this survey as well as affirming that I can withdraw from the survey at any time without giving a reason. The products tested were safe for consumption". The appropriate protocols for protecting the rights and privacy of all participants during the execution of the research were utilized. All the experiments were performed in accordance with relevant guidelines and regulations.

### GC–MS analysis of the volatile organic compounds

The volatile compounds were determined according to previous methods^23,53^. Ground cookie samples (10 g) were precisely weighed into 250 mL quick fit chamber Agricultural Research Service (ARS) (Gainesville, FL, USA). A push–pull Gast pump (Gast Manufacturing Inc., Benton Harbor, MI, USA), was used to pump an activated charcoal-filtered and humidified air over the samples at a flowrate of 340 mL/min as the volatiles simultaneously adhered on GC grade dichloromethane (DCM)-precleaned Super-Q traps (30 mg, Analytical Research System, Gainesville, FL, USA) at 170 mL/min flow rate, sustained by Vacuubrand CVC2 vacuum pump (Vacuubrand, Wertheim, Germany) for 24 h. Trapped volatiles were then eluted with 200 µL of GC-grade DCM (Merck, Darmstadt, Germany) into 250 µL conical point glass inserts (Supelco, Bellefonte, PA, USA) fitted into 2 mL glass vials and immediately queued for GC–MS analysis.

The volatiles were identified by a GC–MS on an HP 7890A series gas chromatograph (Agilent Technologies, Wilmington, NC, USA) attached to an HP 5975C mass spectrometer (Agilent Technologies, Wilmington, NC, USA) operated in electron ionization mode of 70 eV. A non-polar HP-5MS capillary column (30 m 0.25 mm i.d.; 0.25 m film thickness; J & W Scientific, Folsom, CA, USA) was fitted to the instrument. Helium was employed as the carrier gas at a rate of 1.2 mL min^-1^. One microliter of each sample was, in a splitless mode, injected at 35 °C for 5 min, then adjusted to 280 °C at 10 °C min^-1^. The injector and detector were maintained isothermal at 280 °C for 35 min while temperature of the ion source was 230 ℃. Electron ionization mass spectra were recorded at 70 eV spanning a mass range of 38–550 Daltons over a scan period of 0.73 scans s^-1^ (Da). Authentic standard hexanal was run in the GC–MS in full scan mode to generate a linear calibration curve (peak area vs. concentration) with the following equation: $[\mathrm{y }= 203482\mathrm{x}- 451578]$ to yield R^2^ = 0.9997. To identify volatile compounds, their retention periods and mass fragmentation spectra were compared to authentic standards (those available). Others were tentatively identified using Adams, Chemoecol, and the National Institute of Standards and Technology mass spectrum library matching (NIST) (MSD Chemstation E.02.00.493, MS HP, USA). All assays were done in triplicates.

### Statistical analysis

All descriptive and quantitative data were statistically analyzed using R Studio software version 1.3.1093–1^54^. The data sets were verified for normal distribution using the Shapiro–Wilk test (*p* > 0.05). The effects of processing on the digestibility of processed *R. differens* and the related cookies, as well as the distribution of volatile organic components and consumer acceptability of the cookies, were studied using one way analysis of variance (ANOVA). Tukey's and Bonferroni’s multiple comparison tests with *p* < 0.05 were used to differentiate the means. The variations in the sensory scores of the developed cookies were evaluated using Principal Component Analysis (PCA). One-way analysis of similarities (ANOSIM) with the Bray–Curtis dissimilarity matrix was used to examine the chemical profiles of various enriched cookie volatiles. The non-metric multidimensional scaling approach based on the similarity percentages (SIMPER) analysis was used to quantify and illustrate the relative contribution of different compounds to the dissimilarity between volatiles from different cookies. Tabulated results were expressed as mean ± standard deviation.

## Supplementary Information

Supplementary Table S1.

## Data Availability

All relevant data are presented in the paper.
