# Supplementary material for: Aroma characterization and consumer acceptance of four cookie products enriched with insect (Ruspolia differens) meal
Source: Sci Rep. 2023 Jul 10;13:11145. doi: 10.1038/s41598-023-38166-x (PMC10333276; doi:10.1038/s41598-023-38166-x)
Supplement: Supplementary file 1 — Supplementary Table S1. [file 41598_2023_38166_MOESM1_ESM.docx]

**Aroma Characterization and Consumer Acceptance of Four Cookie Products Enriched with Insect (*Ruspolia differens*) Meal**

**Table S1: Volatile organic compounds (µg/g/h) of the cookies enriched with differently processed R. differens analysed by GC-MS.**

| **RT** | **Compound** | **Compound class** | **CTRL** | **BCRC** | **BLRC** | **TSRC** | **DFRC** | **Odour description** |
| --- | --- | --- | --- | --- | --- | --- | --- | --- |
| 3.27 | 3-methyl-Butanal | Aldehyde | 0.23 ± 0.04 |  |  |  | 2.69 ± 0.16 |  |
| 3.9 | Pentanal | Aldehyde | 0.45 ± 0.12 | 0.58 ± 0.11 | 0.75 ± 0.33 | 0.70 ± 0.10 | 3.59 ± 4.39 | nutty, sweet |
| 4.18 | Acetoin | Ketone |  | 0.12 ± 0.01 | 0.66 ± 0.21 | 0.25 ± 0.03 | 0.32 ± 0.04 |  |
| 4.34 | 3-Hydroxy-2-butanone | Ketone | 0.06 ± 0.003 |  |  |  |  |  |
| 4.67 | 2,2,3-trimethyl-Butane | Hydrocarbon |  |  | 0.05 ± 0.001 |  |  |  |
| 4.78 | Pyrazine | Nitrogen compound |  | 0.09 ± 0.02 | 0.11 ± 0.02 | 0.12 ± 0.01 | 0.20 ± 0.03 |  |
| 4.99 | 2-Octen-4-one | Ketone |  |  |  |  | 0.65 ± 0.24 |  |
| 5.00 | (*E*)-3-Penten-2-one | Ketone |  | 0.06 ± 0.01 | 0.31 ± 0.17 | 0.62 ± 0.03 | 0.69 ± 0.03 |  |
| 5.34 | Formic acid pentyl ester | Ester |  |  |  | 0.07 ± 0.01 | 0.09 ± 0.01 |  |
| 5.48 | (2*E*)-Penten-1-al | Aldehyde |  |  | 0.08 ± 0.01 |  |  |  |
| 5.68 | Phenyl ethyl alcohol | Alcohol |  |  |  | 0.41 ± 0.04 |  |  |
| 5.68 | Toluene | Benzenoid | 0.12 ± 0.11 | 0.16 ± 0.14 | 0.09 ± 0.04 | 0.09 ± 0.01 | 0.15 ± 0.09 |  |
| 5.77 | Pentanol | Alcohol | 0.75 ± 0.18 | 0.66 ± 0.28 | 0.86 ± 0.35 | 0.98 ± 0.25 | 1.64 ± 0.17 | plastic |
| 6.17 | 2,3-Butanediol | Alcohol |  | 0.61 ± 0.44 | 0.60 ± 0.36 | 0.73 ± 0.04 | 0.59 ± 0.21 |  |
| 6.37 | 1-Octene | Hydrocarbon | 0.12 ± 0.04 |  |  |  |  |  |
| 6.59 | Hexanal | Aldehyde | 2.60 ± 0.42 | 3.78 ± 1.12 | 5.51 ± 3.43 | 4.82 ± 0.84 | 5.87 ± 3.30 | green apple, grassy |
| 6.95 | 2,3,5-trimethyl-Hexane | Hydrocarbon |  | 0.08 ± 0.01 | 0.15 ± 0.03 | 0.13 ± 0.002 | 0.20 ± 0.01 |  |
| 7.18 | 2,4-dimethyl-Heptane | Hydrocarbon | 0.14 ± 0.02 | 0.12 ± 0.003 | 0.41 ± 0.24 | 0.13 ± 0.002 |  |  |
| 7.18 | Methyl-Pyrazine | Nitrogen compound |  | 2.61 ± 0.20 | 1.80 ± 2.66 | 3.20 ± 1.00 | 5.84 ± 1.33 | nutty, cocoa, roasted meat |
| 7.56 | Furfural | Aldehyde |  | 0.72 ± 0.13 | 0.90 ± 0.30 | 1.00 ± 0.13 | 2.48 ± 0.15 | almond-like, sweet |
| 7.69 | 2,4-Dimethyl-1-heptene | Hydrocarbon |  | 0.21 ± 0.03 | 0.42 ± 0.25 | 0.33 ± 0.04 | 0.82 ± 0.23 |  |
| 8.07 | (2*E*)-Hexenal | Aldehyde |  | 0.38 ± 0.23 |  | 0.37 ± 0.10 | 0.48 ± 0.10 | green, apple-like |
| 8.07 | 2,3-dimethyl-Heptane | Hydrocarbon |  | 0.11 ± 0.02 | 0.19 ± 0.08 | 0.44 ± 0.02 | 0.72 ± 0.04 |  |
| 8.16 | 2-Furanmethanol | Furanoid |  |  | 0.96 ± 0.07 | 0.52 ± 0.03 | 1.63 ± 0.31 |  |
| 8.21 | Ethylbenzene | Benzenoid | 0.48 ± 0.10 | 0.11 ± 0.02 | 0.58 ± 0.14 | 0.42 ± 0.03 | 0.53 ± 0.05 |  |
| 8.25 | 4-methyl-Octane | Hydrocarbon | 0.42 ± 0.16 | 0.73 ± 0.69 | 1.08 ± 0.52 | 1.92 ± 0.17 | 1.64 ± 0.35 |  |
| 8.39 | *p*-Xylene | Benzenoid | 0.31 ± 0.14 | 0.23 ± 0.07 |  | 0.43 ± 0.12 | 0.51 ± 0.26 |  |
| 8.38 | 1,3-dimethyl-Benzene | Benzenoid |  | 0.68 ± 0.12 | 0.44 ± 0.23 |  |  |  |
| 8.47 | 1-Hexanol | Alcohol | 0.54 ± 0.18 |  |  | 0.38 ± 0.11 |  | Fruity, green |
| 8.5 | o-Xylene | Benzenoid |  |  | 0.41 ± 0.12 |  |  |  |
| 8.75 | 2-methyl-Butanoic acid | Carboxylic acid |  |  | 0.58 ± 0.24 |  |  |  |
| 8.9 | 2-Heptanone | Ketone |  | 1.74 ± 0.77 | 2.16 ± 2.52 | 1.29 ± 0.30 | 1.69 ± 0.51 | fruity, sweet |
| 9.24 | Methional | Aldehyde |  |  | 0.73 ± 0.19 | 0.38 ± 0.02 | 0.94 ± 0.06 |  |
| 9.81 | *β*-Phellandrene | Monoterpene |  |  |  | 0.30 ± 0.03 |  |  |
| 9.82 | *α*-Pinene | Monoterpene | 0.59 ± 0.06 | 0.80 ± 0.22 |  | 0.90 ± 0.02 |  | woody, green, pine-like |
| 9.82 | *γ*-Terpinene | Monoterpene |  |  | 0.64 ± 0.28 |  | 1.51 ± 0.29 |  |
| 10.08 | 3,4-Diethyl hexane | Hydrocarbon |  |  |  | 0.35 ± 0.03 |  |  |
| 10.11 | Camphene | Monoterpene |  | 1.10 ± 0.08 | 1.75 ± 0.21 |  | 1.71 ± 0.22 |  |
| 10.11 | 4-methyl-Heptane, | Hydrocarbon | 0.20 ± 0.03 |  |  |  | 0.20 ± 0.03 |  |
| 10.21 | 2,3,4-trimethyl-Decane | Hydrocarbon |  |  | 0.44 ± 0.08 |  | 0.56 ± 0.04 |  |
| 10.35 | (2*E*)-Heptenal | Aldehyde | 0.51 ± 0.15 | 1.30 ± 0.10 | 2.73 ± 0.68 |  |  |  |
| 10.45 | Benzaldehyde | Aldehyde |  | 0.67 ± 0.07 | 1.96 ± 0.43 | 1.57 ± 0.15 | 2.10 ± 0.07 | almond |
| 10.67 | 1-Heptanol | Alcohol |  |  | 0.88 ± 013 |  |  | green |
| 10.71 | *β*-Pinene | Monoterpene | 0.59 ± 0.20 | 0.31 ± 0.05 | 0.72 ± 0.21 |  | 0.93 ± 0.02 | woody, green, pine-like |
| 10.71 | Sabinene | Monoterpene |  | 0.61 ± 0.08 |  |  |  |  |
| 11.01 | 2,2,4,6,6-pentamethyl-Heptane | Hydrocarbon | 0.53 ± 0.19 |  | 0.74 ± 0.24 | 0.39 ± 0.02 |  |  |
| 11.03 | 2-Pentyl furan | Furanoid | 1.36 ± 0.18 | 1.96 ± 1.01 | 4.56 ± 4.48 | 1.96 ± 0.50 | 2.94 ± 0.32 | sweet, woody, almond-like,  baked bread |
| 11.32 | Octanal | Aldehyde | 2.07 ± 1.45 | 0.59 ± 0.09 | 5.22 ± 4.29 | 3.61 ± 0.12 | 4.89 ± 0.18 | fruity |
| 11.3 | *δ*-3-Carene | Monoterpene | 0.50 ± 0.16 |  | 1.92 ± 0.37 | 1.94 ± 0.19 | 1.62 ± 0.30 |  |
| 11.62 | 2-ethenyl-6-methyl-Pyrazine | Nitrogen compound |  |  | 1.12 ± 0.20 |  |  |  |
| 11.79 | Limonene | Monoterpene | 1.01 ± 0.16 | 0.62 ± 0.16 | 0.91 ± 0.29 | 0.63 ± 0.19 | 0.97 ± 0.11 | citrus-like |
| 11.82 | 3-ethyl-2-methyl-1,3-Hexadiene | Hydrocarbon | 1.32 ± 0.28 | 1.07 ± 0.08 | 1.75 ± 0.34 | 1.36 ± 0.12 | 1.57 ± 0.32 | nutty |
| 12.13 | 3-Octen-2-one | Ketone |  |  |  | 0.21 ± 0.02 |  | rose |
| 12.33 | Tricosane | Hydrocarbon | 0.87 ± 0.10 |  | 1.80 ± 0.61 |  |  |  |
| 12.53 | Isoamyl isobutyrate | Ester |  |  |  | 0.68 ± 0.11 |  | green apple |
| 12.55 | (*E*)-2-Octenal | Aldehyde |  | 0.91 ± 0.09 |  | 2.89 ± 0.18 |  | nutty, cooked flour |
| 13.11 | Nonanal | Aldehyde | 7.21 ± 0.57 | 2.08 ± 2.56 | 3.60 ± 1.77 | 4.78 ± 1.68 | 7.44 ± 0.64 | fatty, waxy, pungent |
| 13.77 | trans-3-Nonen-2-one | Ketone |  | 0.51 ± 0.28 | 0.84 ± 0.05 |  | 2.54 ± 0.29 |  |
| 14.08 | (2*Z*)-Nonen-1-al | Aldehyde |  |  | 2.21 ± 0.41 | 0.45 ± 0.03 |  | beany, cucumber |
| 14.56 | Ethyl octanoate | Ester |  |  | 1.64 ± 0.45 |  |  |  |
| 14.77 | Dodecane | Hydrocarbon | 1.79 ± 0.15 | 0.37 ± 0.10 | 0.90 ± 0.20 | 2.55 ± 3.23 | 1.00 ± 0.11 |  |
| 15.66 | (2*E*)-Decenal | Aldehyde |  | 0.39 ± 0.03 |  | 0.57 ± 0.01 | 1.63 ± 0.31 | fatty |
| 15.69 | 4,6-dimethyl-Dodecane | Hydrocarbon |  |  | 0.32 ± 0.02 | 0.22 ± 0.01 |  |  |
| 15.71 | 2,6-dimethyl-Octane | Hydrocarbon |  |  | 0.62 ± 0.26 |  |  |  |
| 15.73 | 2,3,7-trimethyl-Octane | Hydrocarbon |  |  |  |  | 0.28 ± 0.004 |  |
| 15.84 | Tridecane | Hydrocarbon | 1.75 ± 0.23 | 2.00 ± 0.20 | 1.40 ± 0.36 | 0.73 ± 0.01 | 0.53 ± 0.37 |  |
| 16.22 | 2-ethyl-1-Decanol | Alcohol |  |  |  | 0.39 ± 0.03 |  |  |
| 16.4 | Eicosane (C20) | Hydrocarbon |  | 0.19 ± 0.05 | 2.00 ± 0.17 | 0.78 ± 0.07 | 0.40 ± 0.04 |  |
| 16.52 | (2*E*,4*Z*)-Decadienal | Aldehyde |  | 0.64 ± 0.30 | 0.80 ± 0.22 | 1.82 ± 0.76 | 1.23 ± 0.25 | fatty |
| 16.94 | (*E*)-Calamenene | Sesquiterpene |  |  |  |  | 2.36 ± 0.18 |  |
| 17.34 | Propyl butanoate | Ester |  |  | 0.61 ± 0.30 | 1.28 ± 0.14 |  |  |
| 17.5 | Tetradecane | Hydrocarbon | 3.01 ± 0.38 | 1.66 ± 0.21 | 5.14 ± 2.15 | 1.28 ± 0.35 | 1.38 ± 0.96 |  |
| 17.62 | Longifolene | Sesquiterpene | 0.18 ± 0.03 |  |  | 0.28 ± 0.02 |  |  |
| 17.73 | *α*-Cedrene | Sesquiterpene |  |  |  | 0.89 ± 1.14 | 0.35 ± 0.02 |  |
| 18.98 | Butylated hydroxytoluene | Phenylpropane | 0.54 ± 0.02 | 0.44 ± 0.03 | 0.59 ± 0.33 | 0.36 ± 0.16 | 1.34 ± 0.21 |  |
| 20.05 | 2,2,4-Trimethyl-1,3-pentanediol diisobutyrate | Ester |  | 0.41 ± 0.05 |  |  |  |  |
| 26.86 | Docosane | Hydrocarbon |  |  |  | 0.09 ± 0.004 |  |  |
| 26.98 | Octadecane | Hydrocarbon |  | 0.06 ± 0.003 |  | 0.06 ± 0.001 | 0.93 ± 0.04 |  |
| 27.65 | Tetracosane | Hydrocarbon |  |  |  | 0.13 ± 0.01 |  |  |
| Values are presented as Mean ± SD (standard deviation) of triplicate determinations. rT: Retention time; CTRC=Control cookies with eggs; BCRC=Blanched *R. differens*-based cookies; BLRC=Boiled *R. differens*-based cookies; TSRC=Toasted *R. differens*-based cookies; DFRC=Deep fried *R. differens*-based cookies*.* The odour descriptions are retrieved from literature ^43,53–55^. | | | | | | | | |
